# Debulking of a Sarcomatoid Renal Cell Carcinoma: An Unusual Clinical Presentation

**DOI:** 10.7759/cureus.71935

**Published:** 2024-10-20

**Authors:** Alexis Garza, Maria V Rodriguez, David Alonzo

**Affiliations:** 1 Department of Urology, Doctor's Hospital at Renaissance Health, Edinburg, USA

**Keywords:** immunotherapy, metastatic renal cell carcinoma with sarcomatoid features, renal cell carcinoma, sarcomatoid features, urologic oncology

## Abstract

Sarcomatoid renal cell carcinoma (sRCC) is a form of renal cancer known for its aggressiveness and poor prognosis. This report presents the case of a 65-year-old male with an unusual presentation of sRCC. The patient presented with worsening kidney function, flank pain, hematuria, fatigue, and weight loss. Imaging revealed a large, heterogeneous renal mass with evidence of invasion into surrounding structures and lymph nodes. The patient underwent a radical nephrectomy, with the removal of affected structures en bloc, followed by radiation and adjunctive chemotherapy with pembrolizumab. This report discusses challenges in diagnosing sRCC, treatment options, and emerging therapies such as checkpoint inhibitors.

## Introduction

Over 400,000 cases of renal cell carcinoma (RCC) are diagnosed per year worldwide [1]. Sarcomatoid renal cell carcinoma (sRCC) is now considered an undifferentiated carcinoma as opposed to a subtype of sarcoma as previously thought. Considered one of the most aggressive renal cancers, sRCC is known to present late in the disease course and is associated with an extremely poor prognosis. The purpose of this report is to highlight an unusual presentation of sRCC in a 65-year-old male patient and to expand on diagnostics, epidemiology, and treatment options with associated prognostic outlooks.

## Case presentation

A 65-year-old male with a past medical history significant for hypertension (HTN) and chronic kidney disease (CKD) 3/4 secondary to HTN presented to the emergency room (ER), at the discretion of his nephrologist, with an acute kidney injury with a worsening creatinine of 9.7 mg/dL up from a baseline of 3 mg/dL and evaluation of a right renal mass. The patient visited a free-standing ER due to right flank pain onset one week prior in which a computed tomography (CT) of the abdomen and pelvis revealed a right renal mass.

After a thorough review of systems, the patient admitted to a mild right flank pain, a week of slight hematuria, worsening fatigue, nausea/vomiting, and a 10-pound unintended weight loss over the last month. Physical exam was positive for a right upper quadrant (RUQ) abdominal pain and a palpable mass in the right flank. Laboratory results are shown in Table 1. 

**Table 1:** Laboratory values on presentation Hg: hemoglobin; K+: potassium; Na+: sodium; Cr: creatinine; BUN: blood urea nitrogen

	Patient values	Normal range
Hg	9.8 gm/dL	13.5-16.5 gm/dL
Hematocrit	30.3%	40-50%
K+	5.7 mmol/L	3.5-5 mmol/L
Na+	125 mmol/L	132-143 mmol/L
Cr	11.6 mg/dL	0.7-1.3 mg/dL
BUN	97 mg/dL	7-25 mg/dL

The results of a CT urogram showed moderate to severe hydronephrosis in the left kidney due to a possible ureteral stricture. Incidentally, a left inferior renal pole mass was noted, which was described as a lobular exophytic lesion measuring 1.9 cm with no contrast enhancement. Confirmation of a simple renal cyst was done by CT-guided renal biopsy ruling out the possibility of malignancy in his left kidney. Interventional radiology placed a left nephrostomy tube to relieve the hydronephrosis.

**Figure 1:**
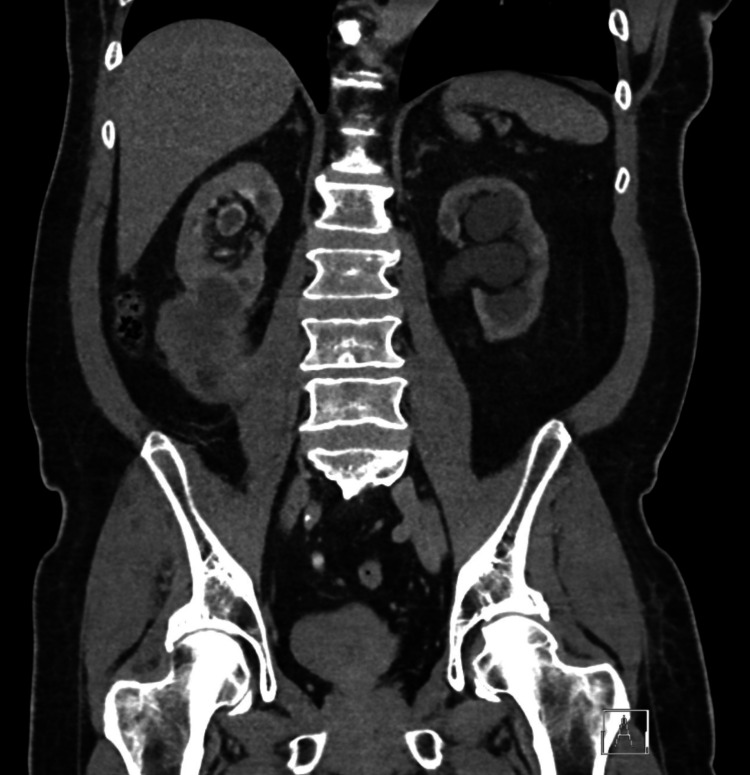
Primary right lower pole renal tumor with involvement with right iliopsoas musculature with filling defect in the superior pelvis on CT urogram CT: computed tomography

**Figure 2:**
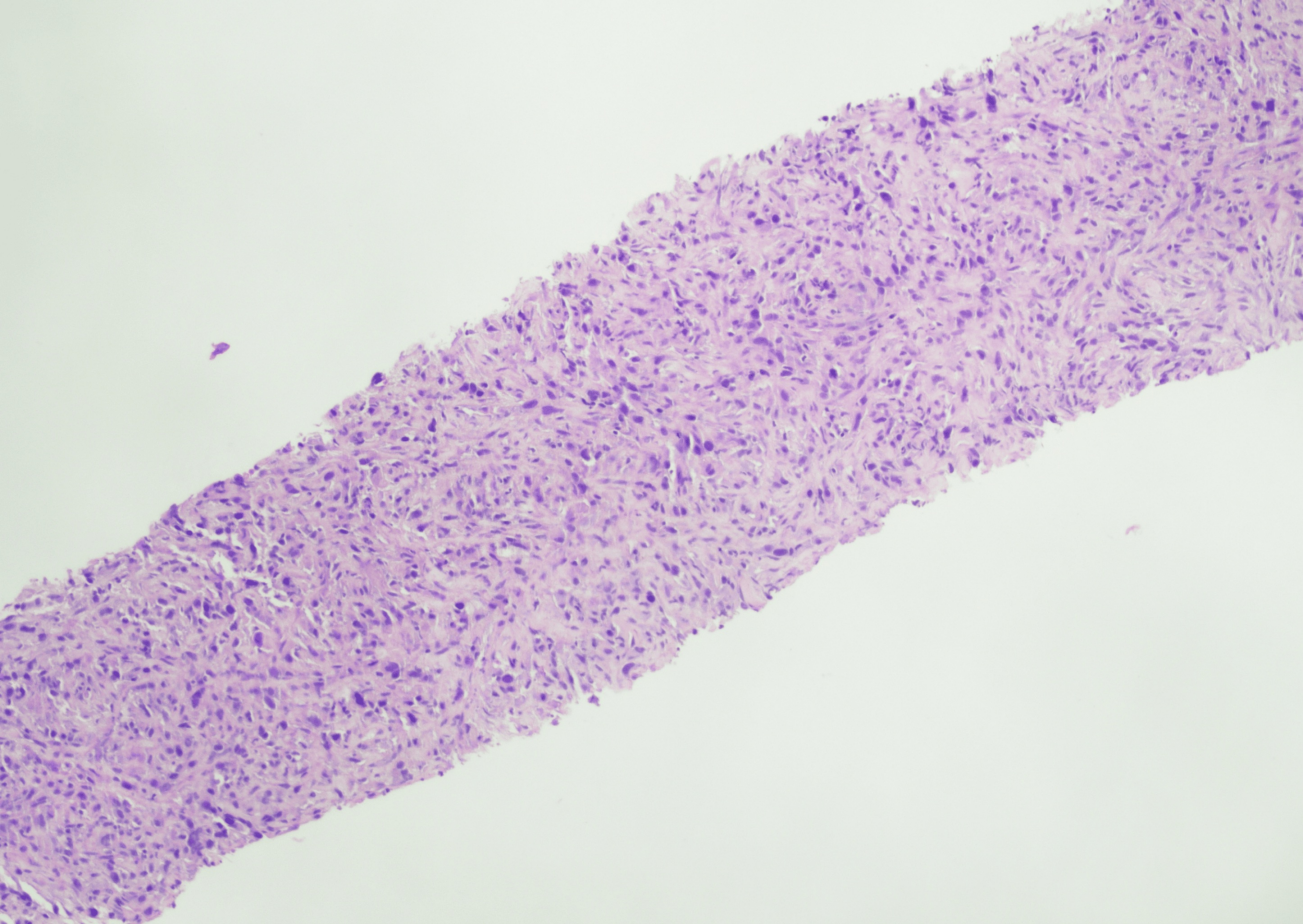
High-grade malignant spindle cell neoplasm on renal biopsy

Concomitantly, the right kidney showed pyelocaliectasis and a large right inferior pole heterogeneous but largely hypoattenuating mass (representing some areas of necrosis) measuring up to 5.4 cm by 6.4 cm by 7 cm anterior-posterior by transverse by craniocaudal. The inferior margin of the mass appeared to abut and invade the outer portion of the right iliopsoas musculature (Figure 1). There were areas of irregular wall thickening noted throughout the right ureter. There were also pathologically enlarged lymph nodes measuring up to 1.2×1.6 cm in the right retrocaval space. CT-guided right renal biopsy was performed by interventional radiology and disclosed evidence of a high-grade malignant spindle cell neoplasm (Figure 2), which presumptively demonstrated a type of sarcoma or sarcomatoid tumor at the level of the kidney. Filling defects were present in the superior pelvis and ureter. Interventional radiology placed a right nephrostomy tube when the right hydronephrosis progressed to prevent further renal injury and alleviate symptoms.

**Figure 3:**
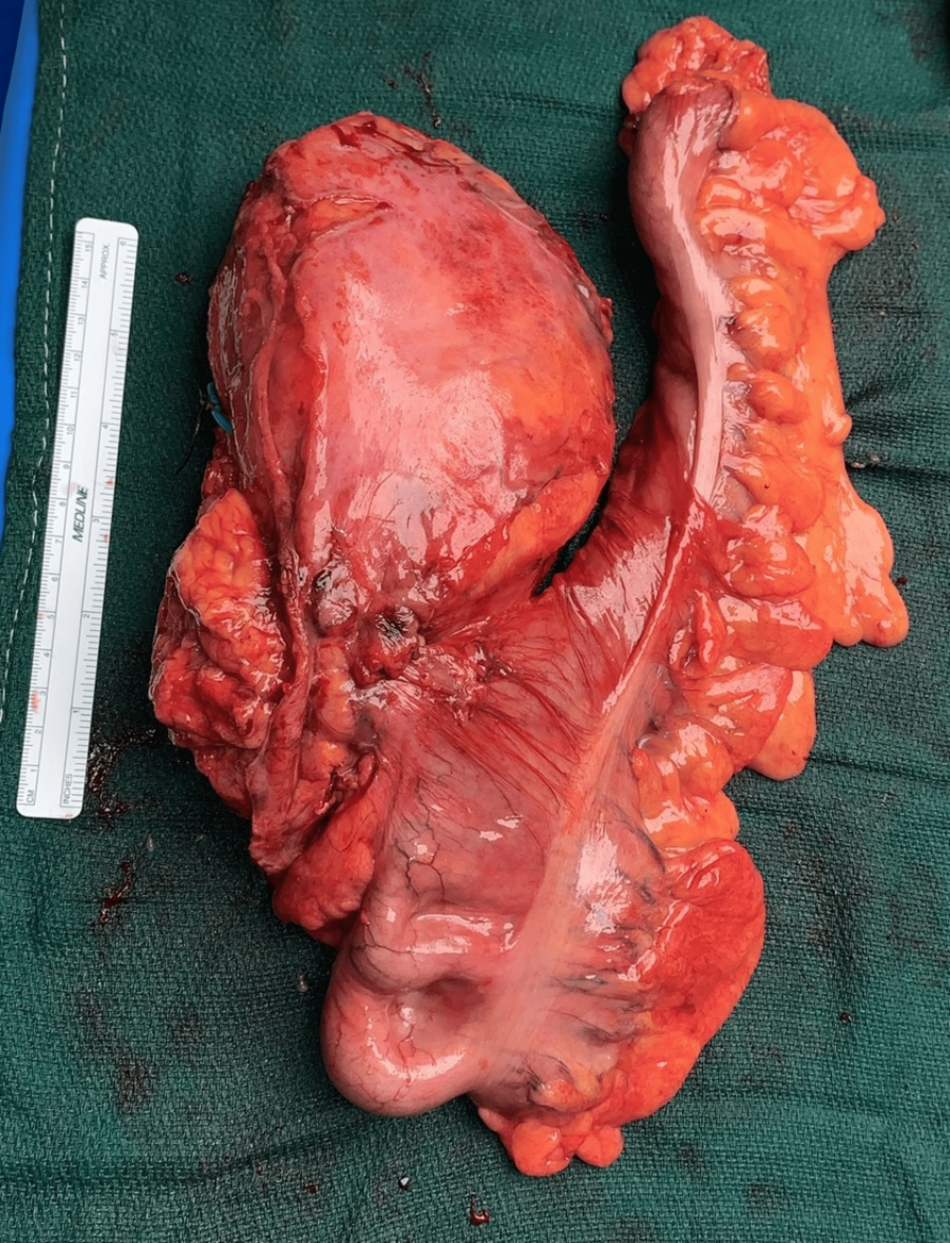
Right kidney specimen irregular in shape with tumor extending past Gerota's fascia. There is invasive extension into the ascending colon, psoas muscle, and genitofemoral nerve, all of which were removed en bloc

**Figure 4:**
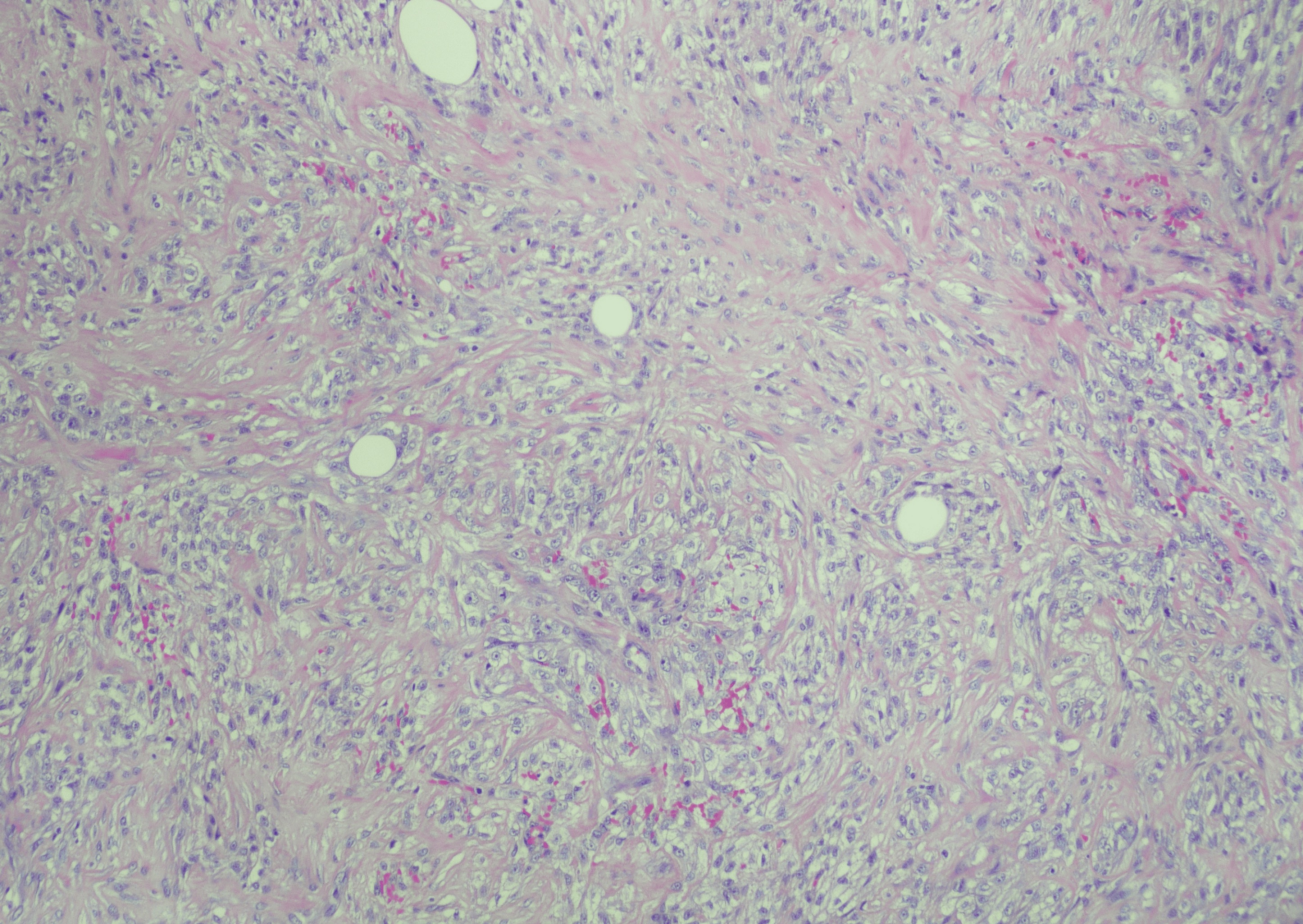
Specimen ISUP grade 4 ISUP: International Society of Urologic Pathologists

**Figure 5:**
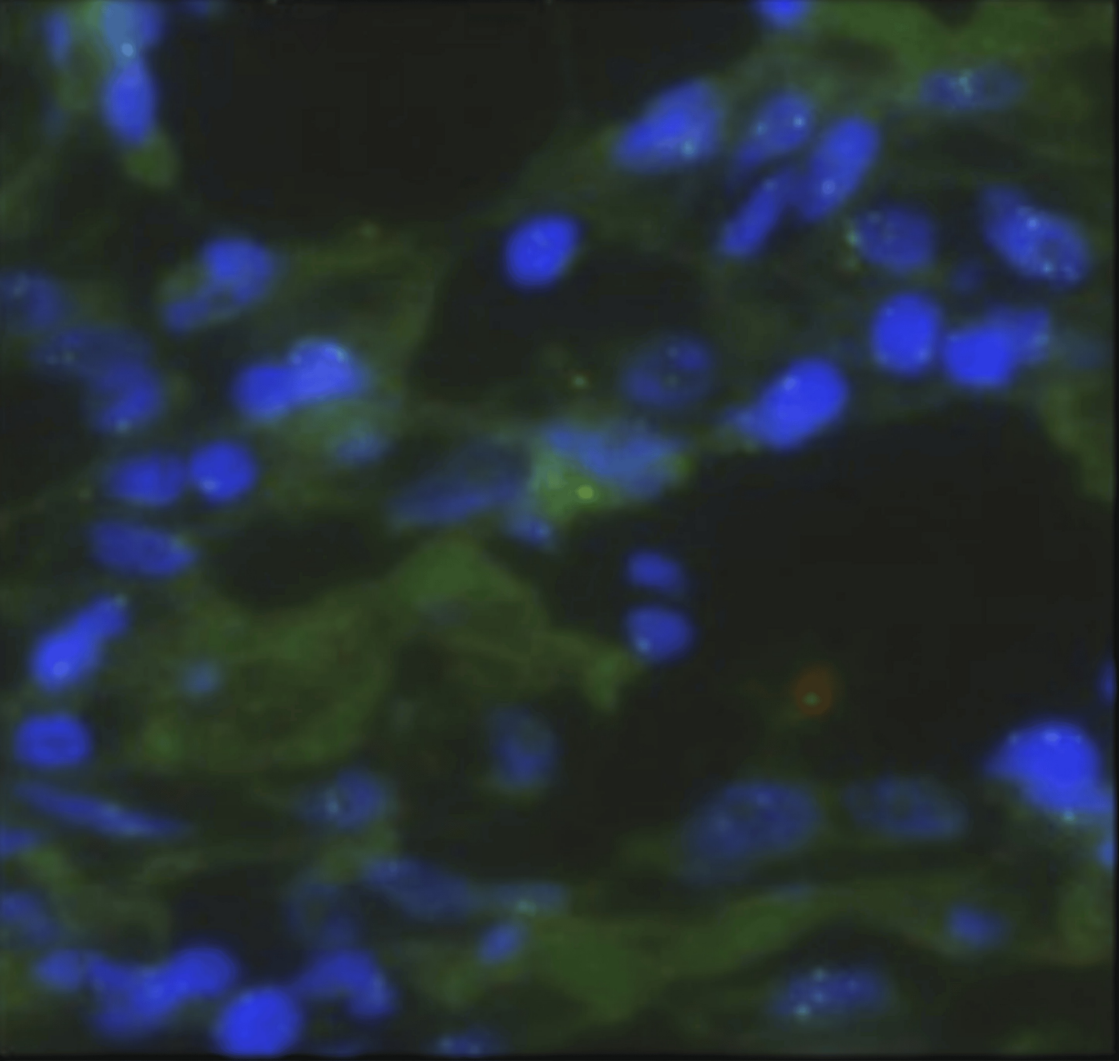
Negative MDM2 FISH FISH: fluorescence in situ hybridization

A right radical nephrectomy was conducted with careful consideration to attain gross negative margins. Upon visualization of the primary tumor located mainly in the inferior pole, it was noted that the tumor extended into most of the retroperitoneum including the right psoas muscle, the genitofemoral nerve posteriorly, and the ascending colon medially. These were subsequently removed en bloc (Figure 3). Various lymph nodes were excised to assess for nodal involvement. The periaortic, iliac, aortocaval, inferior caval, interaorto-caval, and right perihilar nodes (of which were the largest) were removed. The official pathology report was significant for sRCC (International Society of Urologic Pathologists (ISUP) grade 4) in the right kidney (Figure 4), showing signs of necrosis and pericolic fat surrounding the ascending colon, the genitofemoral nerve, the psoas muscle and associated lumbar musculature, and the inferior caval and perihilar lymph nodes (with extension past the nodal capsule). The tumor extended past Gerota's fascia (pT4), with regional lymph node involvement (pN1) and without metastasis confirmed via pan-CT (cM0). There was extensive involvement of soft tissue surrounding the mass, with no involvement in the vasculature, which stained positive for AE1/AE3, CK OSCAR, and PAX-8, and an increased Ki-67 activity. NeoTYPE™ genotype analysis for liposarcoma (NeoGenomics, Fort Myers, Florida, United States) was performed but was negative for MDM2 fluorescence in situ hybridization (FISH) (Figure 5), EWSR1, FUS, HGMA2, and PLAG1 fusions, excluding sarcoma origin. 

The patient was admitted to the intensive care unit for one day and downgraded to the floor on postoperative day 2. He was initially restricted on a diet until he regained bowel function and was progressively advanced to solids on postoperative day 4. There were no postoperative complications, and the patient was instructed to ambulate as soon as he could to prevent thromboembolic events. The patient initiated inpatient rehabilitation for a total of 18 days and commenced radiation therapy and adjunctive immunotherapy with pembrolizumab. He was discharged with a length of hospital stay totaling 53 days. 

## Discussion

RCCs with sarcomatoid features are present in approximately 4-5% of all RCCs, with some studies showing up to 8% incidence [2,3]. This rare transformation equates to an extremely poor prognosis in which most patients rarely live past a year after diagnosis. 

Approximately 90% of patients are symptomatic at first presentation with the most common symptoms including abdominal pain, hematuria, weight loss, and fatigue [4], all of which were experienced by our patient on initial presentation and throughout the hospital course. In close to 50% of cases in one study, metastasis was present with the lung and bone being the most common sites [4]. Another study of a cohort with diagnosed sRCC found that 34% of patients had localized disease, while 66% of patients had some degree of metastasis [5]. A study of close to 900 patients with sRCC found that 60.9% of patients had stage IV disease at the time of diagnosis with a 3.5% five-year disease-specific survival [6]. With such dismal patient outcomes with sRCC, it is of the highest importance to diagnose and treat at the earliest stage of the disease possible. 

Identification of sRCC through imaging and needle biopsy has proven tedious due to the nonspecific nature of imaging and the low sensitivity of needle biopsies [7]. Imaging of possible sRCC has relied on the cardinal features of the tumor itself: large tumor size, the presence of neovascularity, and invasive tumor margins [8]. Ongoing studies are been conducted in which more sophisticated imaging techniques can differentiate sRCC from other tumors of the kidney which could pave the way to shortening the time to diagnosis and help patient outcomes [8,9]. Efforts have been made to increase the sensitivity of needle biopsies as confirmation can aid greatly in surgical approaches and improve treatment modalities in advance. One study shows that implementing "multi-quadrant sampling" could push the sensitivities of biopsies closer to 90% [10]. 

Histologically, sRCC contains similar aspects to sarcomas. The biopsy obtained prior to nephrectomy disclosed "high-grade malignant spindled-cell neoplasm," leading us to believe that the mass was sarcoma in origin. The differentiating factor in sRCC is the presence of carcinoma elements in addition to the spindle cells that range from 1% to 99% with a mean of ~45% [11]. As mentioned before, even though sRCC is not a subtype of RCC and could arise from each subtype of RCC, the pathologist must make the distinction due to the prognosis and the effect on choosing treatment modality. This has become a standard of those in the genitourinary surgical pathology field in which there is no explicit minimum percentage of sarcomatoid feature in a specimen in order to diagnose an sRCC [12]. 

Special attention is taken in order to differentiate sRCC from that of renal sarcomas. For one, primary renal sarcomas are extremely rare, encompassing less than 1% of all renal tumors. The most common sarcoma of the kidney is leiomyosarcoma which expresses smooth muscle components that are rarely observed in tumors of epithelial origin. To further differentiate, special stains may be used. Regarding immunohistochemical staining, most sRCC stain positive for AE1/AE3, vimentin, CK OSCAR, PAX-8, and epithelial membrane antigen owing to the epithelial origin of the cancer [13,14]. The presence of AE1/AE3 is present in about 97% of carcinomas which differs from their sarcoma counterparts that mainly express desmin and actin, two stains that are predominantly negative in carcinomas [15]. PAX-8 is a "master regulator" of gene expression during kidney development and has been found to be increased in renal carcinomas as in our patient [13]. 

In some studies, the presence of sarcomatoid differentiation in RCC patients increased the risk of death between 58% and 82% depending on multiple variables such as the presence of distant metastasis and the percent of the total tumor with differentiation [16]. One of the major differentiating factors leading to poor prognosis is the presence of late-stage disease at the time of presentation. In the case of our patient, evidence of a renal mass was established a month prior to presentation in the ER. The extension of the primary tumor past the renal capsule and Gerota's fascia in addition to the extension into surrounding psoas musculature adds to poor prognosis even without distant metastasis. This partnered with the presence of necrosis in areas with sarcomatoid dedifferentiation in our patient has been shown to further the poor prognosis [17].

The realm of treatment for sRCC is limited due to the late stage of the disease at the time of diagnosis. Because partial nephrectomy is usually insufficient in removing the large and bulky nature of sRCC [15], the initial treatment modality is usually radical nephrectomy. This is potentially curative for localized RCC, and the outcomes for localized sRCC are subpar. In one study of 73 patients with no evidence of disease following the resection of sRCC, 77% experienced recurrence with a median time of recurrence of 26.2 months [18]. This recurrence might be due to the extension of the primary tumor into adjacent structures, which further complicates resection, or to regional lymph nodes. Researchers at the Mayo Clinic recommend an extended dissection of lymph nodes if the primary cancer is positive for sarcomatoid features [19], as was the case in our patient. Even with significant resection however, morbidity and mortality are rarely lessened. 

In addition to surgical resection, systemic therapy may be added on to reduce morbidity and mortality. Prior to the initiation of anti-vascular endothelial growth factor (anti-VEGF) agents in 2007, therapeutic options for sRCC were limited to chemotherapeutic agents that target DNA synthesis such as doxorubicin and gemcitabine. The results of a phase II study combining both these agents showed an objective response rate (ORR) of only 16% and a complete response rate of 2.7%, with a median overall survival (OS) of 8.8 months and a median progression-free survival (mPFS) of 3.5 months [20]. Agents targeting VEGF receptors, such as sunitinib and axitinib, were introduced which improved outcomes in patients with sRCC, albeit marginally. Four different trials had sunitinib ORR ranging from 14% to 31.5% and OS ranging from 14.2 to 15.4 months with a mPFS of 4-8.4 months [21]. Another study in which patients were split into two groups, good and poor performance status, based on the patient's ability to perform activities of daily living found that the median survival was 20.9 months in patients with good performance status but decreased to five months in those with poor performance status [22].

In recent years, much attention has been paid to checkpoint inhibitors as a modality to treat sRCC. It has been shown that sRCC is known to highly express PD-1/PD-L1 on the cell, receptors pivotal to the evasive nature of tumor cells [23,24]. One study found positive PD-L1 expression in 89% of their sRCC samples with 96% PD-1 expression in tumor-infiltrating lymphocytes [25]. With this information, new checkpoint inhibitors have been formulated to tackle these overexpressed targets. Pembrolizumab and nivolumab target PD-1 while atezolizumab and avelumab target PD-L1 to minimize this interaction thought to aid in tumor immune evasion. In numerous trials, a combination of checkpoint inhibitors and anti-VEGF therapy has substantially increased survival in patients with sRCC [20,26-28]. In the KEYNOTE-426 intention-to-treat cohort, pembrolizumab plus axitinib showed the highest efficacy of any combination treatment with an ORR of 59% compared to just 32% with sunitinib alone [29]. In the 112-patient CheckMate 214 trial focusing on the International Metastatic Renal Cell Carcinoma Database Consortium (IMDC) intermediate- and poor-risk group, nivolumab plus ipilimumab, a CTLA-4 binder, showed improved efficacy with an ORR of 57% compared to just 19% with sunitinib alone [30]. Combination therapy utilizing checkpoint inhibitors is a hot topic in current research with multiple clinical trials racing to advance treatment options. 

The role of radiation therapy in RCC has been unclear in the past. Many regarded RCC as a "radioresistant" tumor, deeming this therapy futile. Its main role has solely been in the area of distant metastasis, such as tumor-infiltrating bone and brain, and mainly in a palliative sense. A meta-analysis in 2010 to assess the efficacy of postoperative radiotherapy (POST) was conducted to see if radiation had any effect on the OS, disease-free survival (DFS), and locoregional failure (LRF). In this analysis of 735 total patients with RCC treated with POST, there was a statistically significant decrease in LRF (odds ratio: 0.476; 95% CI: 0.334-0.680) with no significant change in OS or DFS [31]. Rodriguez-Fernandez et al. found similar results with a meta-analysis of 12 studies encompassing 1624 patients [32]. This consensus seems to be dated to the time period before the advent of high-dose-per-fraction treatment techniques, as new studies lead us to believe [33,34]. This combined with leading-edge immunotherapy options seems to be yielding promising preclinical and clinical trial results, but this is still an area of ongoing research [35].

## Conclusions

Although strides have been made in the diagnosis and treatment of sRCC, concrete guidelines in these areas have not been formulated. Much of this is due to the ongoing research to better understand this pathology. With promising new diagnostic and treatment options, morbidity and mortality will continue to decrease with a hopeful increase in quality of life and OS. With the advent of immunotherapy such as checkpoint inhibitors and the advancement of radio-diagnostics and radiotherapy, diagnosing sRCC early with the added benefit of tackling the pathology from numerous angles will prove beneficial in the future. 
